# Evaluating the effect of mechanical debridement with adjunctive antimicrobial photodynamic therapy in comparison with mechanical debridement alone on the peri-implant parameters in type 2 diabetic mellitus patients with peri-implantitis: a systematic review and meta-analysis

**DOI:** 10.1186/s12903-023-03337-9

**Published:** 2023-10-12

**Authors:** Shima Afrasiabi, Mohadeseh Heidari, Shima Younespour, Nasim Chiniforush

**Affiliations:** 1 Laser Research Center of Dentistry, Dentistry Research Institute, Tehran University of Medical Sciences, Tehran, Iran; 2 Dental Implant Research Center, Dentistry Research Institute, Tehran University of Medical Sciences, Tehran, Iran; 3 Dentistry Research Institute, Tehran University of Medical Sciences, Tehran, Iran; 4 Department of Surgical Sciences and Integrated Diagnostics, University of Genoa, Genoa, Italy

**Keywords:** Antimicrobial photodynamic therapy, Adjunctive methods, Peri-implantitis, Mechanical debridement, Diabetes mellitus, Systematic review, Meta-analysis

## Abstract

**Background:**

Type 2 diabetes mellitus (T2DM) is a major risk factor for localized diseases such as peri-implantitis that may affect ideal implant treatment. This study was aimed to evaluate the effect of mechanical debridement (MD) + antimicrobial photodynamic therapy (a-PDT) in patients with peri-implantitis who have T2DM in terms of bleeding on probing (BOP) and probing depth (PD) as primary outcomes and plaque index (PI) and crestal bone loss (CBL) as secondary outcomes.

**Methods:**

Publications compared outcomes between MD + aPDT and MD alone in T2DM patients with peri-implantitis, containing more than 3-month follow-up duration, were involved in the systematic review and meta-analysis. Literature until July 2023 using MEDLINE (through PubMed), Scopus, Cochrane Library, Embase, Web of Science, and Google Scholar were collected.

**Results:**

Two randomized controlled trials (RCTs, 88 individuals) and one controlled clinical trial (CCT, 67 individuals) with follow-up periods ranged from 3 to 12 months were recruited. All studies used diode laser with wavelengths ranged from 660 to 810 nm. The results demonstrated that the MD + aPDT group showed significant benefits for BOP reduction after 6 months (SMD = -2.15, 95% CI: -3.78 to -0.51, p = 0.01). However, a great amount of heterogeneity was observed (*I*^*2*^ *=* 91.52%, p < 0.001). Moreover, there was a significant difference between MD + aPDT and MD alone groups in CBL (SMD = -0.69, 95% CI: -1.07 to -0.30, p < 0.001). In addition, homogeneity assumption was satisfied (*I*^*2*^ = 22.49%, p = 0.28). Significant differences in PD and PI reduction were not found except for PI reduction after 3 months (SMD = -0.79, 95% CI: -1.24 to -0.33, p < 0.001. Also, no heterogeneity was observed (*I*^*2*^ *=* 0.00%, p = 0.47).

**Conclusion:**

Given that high heterogeneity in BOP and PD outcome was found in this systematic review, future long-term CTs with MD + aPDT should be examined to arrive at a firm conclusion.

**Supplementary Information:**

The online version contains supplementary material available at 10.1186/s12903-023-03337-9.

## Introduction

Peri-implantitis is an inflammatory process affecting the tissues surrounding osseointegrated dental implants and results in pocket formation, purulence, and loss of the supporting bone, which is associated with the reduction of implant survival [1]. The mean prevalence of peri-implantitis has been reported 19.83% [2]. The number of inserted implants is expected to increase every year due to the success rate of implant therapy and the increase in the elderly population in the world [3]. In a prospective cohort study, the implant survival rate was 91.6% and showed 7% peri-implantitis after 10 years of follow-up [4].

Peri-implantitis is a plaque associated disease [5]. Currently, there are surgical and non-surgical treatments for peri-implantitis. Surgical treatment of peri-implantitis is often associated with high morbidity for the patient [6, 7]. Mechanical debridement (MD) involves the use of supra- and subgingival debridement of the implant/ abutment surface. The main objective is an effective removal of biofilm and calculus from the implant surface with the aim of restoring a healthy mucosa around the implants [8]. Surgical and nonsurgical MD can improve important clinical parameters, but the amount of bacteria does not decrease to undetectable levels and there is not yet a gold standard protocol [9]. Moreover, this treatment method does not seem to be effective in moderate to severe lesions of peri-implantitis [10]. Studies have reported that instruments used in the nonsurgical method may crack the implant surface, and prevent complete healing of the implant prosthesis [11]. In addition, these instruments cannot be used in deep pockets or areas that are anatomically complex [12].

Antimicrobial photodynamic therapy (a-PDT) consists of three fundamental components: light source, photosensitizer, and oxygen. After activation of a photosensitizer and release of free radicals, the cell wall of pathogens is destroyed [13]. One of the advantages of a-PDT as a cost-effective treatment compared to other treatments is that it is noninvasive with significant destruction to microorganisms [14]. According to the study of Al-Askar et al. the use of aPDT offers a significant improvement in reducing probing depth (PD), bleeding on probing (BOP) and plaque index (PI) in conjunction with the conventional approach [15].

The number of people with Type 2 diabetes mellitus (T2DM) is increasing in all regions of the world [16]. T2DM disease shows evidence of dysregulation of macromolecules, including carbohydrate, protein, and lipid metabolism, resulting in insulin resistance, impaired insulin secretion, or a combination of both [17]. Poorly controlled T2DM is a risk factor for oral diseases, including mouth dryness, taste disturbance, caries, fungal infections, periodontal disease, and peri-implantitis [18–20]. Due to the high prevalence of T2DM, the demand for implant treatments is increasing as T2DM manifests itself through tooth loss [21]. It has been shown that implant therapy can be a safe treatment option when T2DM is well controlled [22]. However, it is known that patients with uncontrolled T2DM have an increased risk of delayed recovery, microvascular complications, tissue damage, and infection, which can impair and compromise implant bone integrity, leading to increased treatment failure [23]. aPDT has also been reported to help improve clinical and antimicrobial parameters in T2DM patients [24].

With regards to the benefits of a-PDT, its efficacy as an adjunct in improving clinical periodontal parameters in T2DM is unknown. The present study aimed to assess the effect of a-PDT as an adjunctive treatment to MD in clinical outcomes in T2DM with peri-implantitis.

## Materials and methods

The current systematic review was conducted in accordance with the statement of preferred reporting items for systematic reviews and meta-analyses (PRISMA) [25].

### Definitions of peri-implantitis

Peri-implantitis is characterized by radiographic evidence of loss of crestal bone loss (CBL) coupled with peri-implant soft tissue inflammation [26]. Case definitions for peri-implantitis often use clinical signs of inflammation such as redness, edema, mucosal enlargement, positive BOP, suppuration, increased PD and radiographic evidence of bone loss [27].

### Focused question

The addressed focused question was: “Does a-PDT as an adjunctive treatment with MD improve clinical outcomes in terms of BOP, PD, CBL, and PI as a hygiene parameter in the treatment of peri-implantits in patients with T2DM?”

### Inclusion and exclusion criteria

#### Types of studies

Randomized controlled trials (RCTs), controlled (non-randomized) clinical trials (CCTs) were included in the current study. Non-CCTs, one group before-after trials, prospective and retrospective cohort studies, case series, case reports, narrative literature reviews, pilot studies, animal studies and letters to the editor were excluded.

#### Participants

Studies treating adult individuals aged 18 years and above with peri-implantitis who are experiencing T2DM (A1C ≥ 6.5%) were included [28]. There were no restrictions regarding gender, race, ethnicity, language, publication date, photosensitizer, and light source parameters.

#### Intervention

The intervention group in the included studies was a-PDT adjunct to MD. Studies with less than three months of follow-up were excluded.

#### Comparison

The comparison group in the included studies was MD treatment alone.

#### Types of outcome measures

##### Primary outcomes

BOP and PD at six sites around each implant (mesiobuccal, mesiopalatal/lingual, midbuccal, midpalatal/lingual, distobuccal, distopalatal/lingual) at baseline and the available time points were considered as primary outcomes. PD was defined as the distance from the gingival margin to the base of the periodontal pocket using a periodontal probe. BOP was defined as the percentage of sites with marginal bleeding on gentle probing using a periodontal probe. Studies with at least one of the outcome variables were included.

##### Secondary outcomes

Secondary outcomes were PI and CBL at baseline and the available follow-ups. CBL in millimeters (mm) was defined as the vertical distance between the baseline peri-implant bone level at mesial and distal and follow-up time point.

### Information sources and search strategy

Medline (through PubMed), Scopus, Embase, Web of Science and Cochrane Library electronic databases were searched up to 20th July 2023 to address the focused question. Furthermore, Google Scholar, ClinicalTrials.gov and WHO International Clinical Trial Registry Platform search were performed. Open grey search was conducted to find gray literature. The reference lists of included studies were manually searched. In addition, hand searching was performed for key journals including Journal of Clinical Periodontology, Journal of Periodontology, Journal of Esthetic and Restorative Dentistry, International Journal of Periodontics and Restorative Dentistry, Photodiagnosis and Photodynamic Therapy, Journal of Photochemistry and Photobiology B, Journal of Lasers in medical sciences, Journal of Lasers in medical science, and Photobiomodulation, Photomedicine and Laser Surgery.

MeSH and Emtree databases were searched to identify Medical Subject Headings (MeSH) and Embase Subject Headings (Emtree), respectively. Entry terms in MeSH and synonyms in Emtree and word variants were used to add additional keywords to search syntax. Search syntax for PubMed is presented in Table S1 [29]. S.A, M.H, and S.Y developed the search strategy and S.A, and S.Y performed the search.

### Study selection

All studies retrieved from electronic and hand searches were entered into Endnote x8. After duplicates removal, two reviewers (S.A and N.C) separately carried out the two-step screening, with studies screened via titles and abstracts followed by full-text review considering inclusion and exclusion criteria. In case of missing full-text, we contacted the authors via email to obtain the full-text. All studies meeting the inclusion criteria included in the current systematic review. A third reviewer (S.Y) resolved any disagreements that could not be resolved by consensus of the first two reviewers.

### Data extraction

Data extraction was performed in duplicate (S.A and S.Y) via standardized data extraction tables, independently. Conflicts were resolved by consensus discussion. Extracted data were as follows: Authors’ name, publication date, study design, country, follow-up duration, eligibility criteria, status of blinding, allocation concealment, and other concerns about bias, demographic characteristics of study individuals such as age and gender, number of individuals per each group, type of periodontal treatment (adjunctive a-PDT to MD, MD alone), laser parameters including type of laser, wavelength, energy fluence, power density, duration of irradiation, optic fiber diameter, type of photosensitizer, pre-irradiation time, concentration of photosensitizer, number of laser sessions, primary outcomes (BOP and PD) and secondary outcomes (PI and CBL) at baseline and each of the follow-up time points and measure of effects. We contacted the authors of included studies via email to provide incomplete information.

### Risk of bias assessment

Double blinded authors (S.A and S.Y) conducted the risk of bias assessment of the included articles, individually. Any disagreement was resolved by discussion with a third reviewer (M.H). The Cochrane Handbook for Systematic reviews of Interventions and the CONSORT statement guidelines were used to determine the quality of the included clinical trials [30, 31]. The total judgment was as follows: low risk of bias (if all the domains were determined as low risk of bias); unclear risk of bias (if at least one item was considered as unclear risk of bias); or high risk of bias (if at least one item was judged as high risk of bias).

### Statistical methods

Mean ± SD was used to summarize the study outcomes. Review Manager (RevMan) [Computer program]. Version 5.4.1, The Cochrane Collaboration, 2020 was used to illustrate the risk of bias in the included studies. Meta-analyses were conducted on primary outcomes (BOP and PD) and secondary outcomes (PI and CBL using STATA/MP 16.0. To assess the statistical heterogeneity among the included studies, χ^2^ and I^2^ statistics were applied. Weighted mean difference (WMD) and standardized mean difference (SMD) of outcomes with 95% confidence intervals (CI) at 3-month and 6-month follow-up was graphically presented via forest plots. Due to small number of included studies, no sub-group analysis could be performed to explore the source of heterogeneity.

## Result

### Study selection

**Fig. 1:**
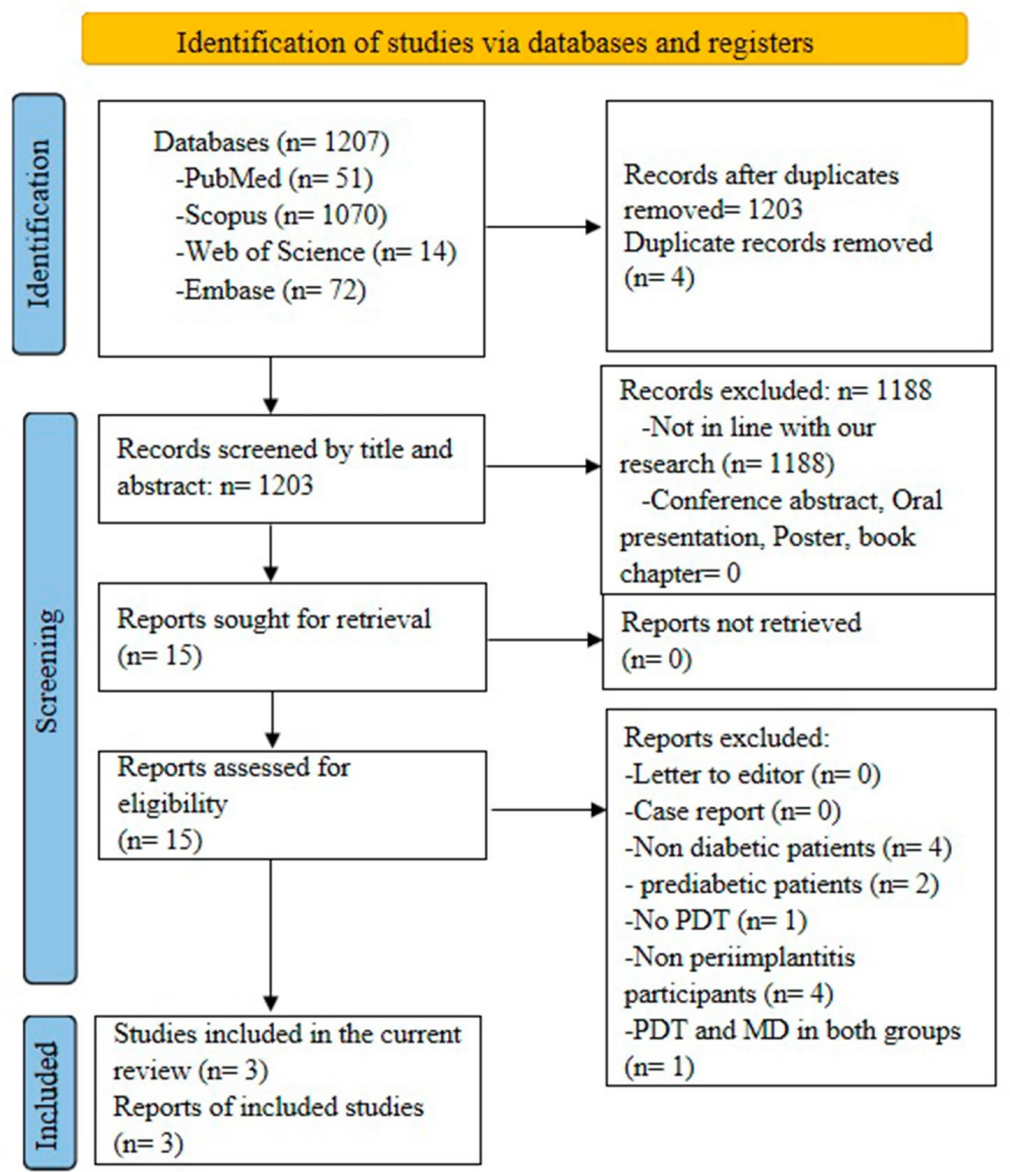
PRISMA flowchart

As illustrated in the PRISMA flow diagram (Fig. 1), the search strategy retrieved 1207 records. After duplicates’ removal, 1203 references were remained. By screening the titles and abstracts, 1188 articles were excluded due to unrelated topic. Then, the remaining 15 articles were assessed and 3 articles met the pre-specified eligibility criteria and were considered for data extraction.

### General characteristics of included studies

Two RCTs (88 individuals) and one CCT (67 individuals) met the inclusion criteria and included in the current systematic review [23, 32, 33]. Characteristics of the included studies are summarized in Table 1. The included studies originated from Saudi Arabia and were in English language with publication dates ranging from 2016 to 2021. The mean sample-weighted age of patients was 50.76 years. There were 100 males and 55 females. The mean sample-weighted duration of T2DM was 10.78 years [23, 32, 33]. The criteria for diagnosing peri-implant diseases were different among the included studies. Al Amri et al. study [23] included participants with PD ≥ 4 mm and BOP in at least 30% sites diagnosed as peri-implantitis, while other studies included individuals with peri-implant diseases characterized with PD ≥ 6 mm with BOP on at least one site around dental implants [32, 33]. All included studies used MD + a-PDT as intervention group and MD alone as comparison group. The follow-up duration of included articles ranged from 3 to 12 months.

**Table 1:** Characteristics of included studies

**Author, Year;** **country**	**Sample size; Age; Male/female ratio; Duration of T2DM**	Diagnostic criteria for peri-implant disease*/ **Diagnostic criteria for T2DM**	**Primary outcomes**								**Secondary outcomes**										
			**Bleeding On Probing (%)**				**Probing Depth (mm)**				**Plaque Index (%)**				**Crestal bone loss (mm)**						
			Baseline	M3	M6	M12	Baseline	M3	M6	M12	Baseline	M3	M6	M12	Baseline	M3	M6		M12		
Al Amri, et al., 2016; Saudi Arabia	**MD** **n**:33; **Age**: 51.4 ± 3.7; **M/F**:17/16 **T2DM duration**: 10.5 ± 0.2	Patients with peri-implantitis (peri-implant BOP in at least 30% of sites, and CBL ≥ 2 mm Patients with medically diagnosed T2DM (HbA1c level ≥ 8%)	31.7 ± 9.4	-	15.1 ± 3.4	10.5 ± 1.3	19.5 ± 2.4^#^	-	8.5 ± 1.4^#^	4.3 ± 0.7^#^	N/A	N/A	N/A	N/A	1.3 ± 0.6	-	1.4 ± 0.1		1.3 ± 0.1		
	**MD + aPDT** **n**:34; **Age**: 53.6 ± 9.5; **M/F**: 19/15 **T2DM duration**: 11.2 ± 2.7		36.3 ± 14.2	-	9.2 ± 1.6	2.4 ± 0.6	16.2 ± 3.7^#^	-	3.1 ± 0.8^#^	0.4 ± 0.1^#^	N/A	N/A	N/A	N/A	1.4 ± 0.2	-	1.3 ± 0.1		1.3 ± 0.2		
Ahmed, et al., 2020; Saudi Arabia	**MD** **n**:20; **Age**: 50.7 ± 5.9; **M/F**:20/0 **T2DM duration**: 8.9 ± 1.5	Patients with mild BOP; CAL ≤ 3 mm; PD ≥ 6 mm on at least one dental implant site; ≥ 3 mm alveolar bone loss apical to the coronal region of the intraosteal portion of dental implant; Patients with medically diagnosed T2DM (HbA1c level ≥ 6.5%)	46.6 ± 24.3	27.7 ± 12.6	19.8 ± 9.4	-	6.9 ± 1.8	5.4 ± 1.7	4.7 ± 1.1	-	44.2 ± 10.6	22.3 ± 8.4	14.2 ± 6.5	-	1.8 ± 0.5	1.7 ± 0.4	1.2 ± 0.7		-		
	**MD + aPDT** **n**:20; **Age**: 48.9 ± 4.5; **M/F**: 20/0 **T2DM duration**: 9.4 ± 3.1		52.6 ± 24.6	21.3 ± 12.6	14.5 ± 6.7	-	6.8 ± 1.4	4.3 ± 0.9	4.0 ± 0.9	-	43.3 ± 8.6	17.8 ± 4.6	13.7 ± 6.8	-	1.9 ± 0.4	1.6 ± 0.2	1.0 ± 0.6		-		
Labban, et al., 2021; Saudi Arabia	**MD** **n**:24; **Age**: 50.4 ± 9.3; **M/F**:11/13 **T2DM duration**: 11.7 ± 4.6	Patients with PD ≥ 6 mm with bleeding on at least one site around dental implants; ≥ 3 mm alveolar bone loss apical to the coronal region of the intraosteal portion of dental implant; Patients with medically diagnosed T2DM (HbA1c level ≥ 6.5%)	63.65 ± 11.34	42.62 ± 12.15	31.58 ± 15.89	-	6.62 ± 0.35	5.17 ± 0.79	4.93 ± 0.87	-	57.91 ± 9.77	20.45 ± 6.31	17.06 ± 4.01	-	3.95 ± 1.16	2.19 ± 1.42	1.92 ± 1.13		-		
	**MD + aPDT** **n**: 24; **Age**: 47.8 ± 7.2; **M/F**: 13/11 **T2DM duration**: 12.4 ± 3.9		59.12 ± 9.37	21.34 ± 6.78	19.19 ± 5.98	-	6.47 ± 0.52	4.93 ± 0.88	3.81 ± 0.54	-	53.98 ± 15.18	17.34 ± 4.83	15.19 ± 3.76	-	3.82 ± 1.09	2.06 ± 1.13	1.31 ± 0.95		-		

# PD ≥ 4 mm was measured at 6 sites per implant and presented as mean percentage per individual.

Abbreviations: MD, Mechanical Debridement; aPDT, antimicrobial Photodynamic Therapy; CBL, Crestal Bone Loss; BOP, Bleeding On Probing; PD, Probing Depth; N/A, Not Available.

### a-PDT-related parameters

Table 2 presents a-PDT related parameters of included studies. All studies used diode laser with wavelengths ranging from 660 to 810 nm [23, 32, 33]. Energy fluence and optic fiber diameter were reported by none of the included studies. The output power ranged between 100 and 200 milliwatts [23, 32, 33]. Power density was mentioned in one study which was 1.1 W/cm^2^ [32]. Pre-irradiation time was 120 s in two studies [23, 32] and was not mentioned in the Labban et al. study [33]. The duration of irradiation was 10 s each pocket in two studies [23, 32] and 50 s total in the Labban et al. study [33]. Phenothiazine chloride was used as photosensitizer in two studies [23, 32] and indocyanine green was used by the other study [33]. The concentration of photosensitizer was reported by two studies which was 0.005% [32] and 1 mg/mL [33].

**Table 2:** Summary of laser and photosensitizer parameters of included studies

Authors, years	Type of Laser	Wavelength (nm)	Energy fluence (J/cm^2^)	Power output (mW)	Power density (W/cm^2^)	Duration of irradiation (seconds)	Optic fiber diameter (mm)	Type of PS	Pre-irradiation time (seconds)	Concentration of PS	Number of laser sessions	
Al Amri, et al., 2016	Diode laser	660	N/A	100	N/A	10 s each pocket	N/A	PTC	120	N/A	1	
Ahmed, et al., 2020	Diode laser	660	N/A	150	1.1	10 s each site	N/A	PTC	120	0.005%	1	
Labban, et al., 2021	Diode laser	810	N/A	200	N/A	50 s total	N/A	ICG	N/A	1 mg/mL	4	

Abbreviations: PS; photosensitizer; PTC, phenothiazine Chloride; ICG, Indocyanine green; N/A; Not Available, nm; nanometers; J/cm^2^; joules per square centimeters,

mW, milliwatts; W/cm^2^; Watts per square centimeters

### Risk of bias

Figure 2 illustrates the risk of bias in the included studies. Judgments of review authors (S.A and S.Y) about each risk of bias item for included studies are reported in Fig. 3. In two CTs, participants were randomly divided into two groups using block randomization [32, 33]. However, randomization was not performed in Al Amri et al. study [23]. We judged the level of risk to be low in two studies [32, 33] and to be high in Al Amri et al. study [23]. Allocation concealment was not performed in Al Amri et al. study and we rated this study as high risk of bias [23]. Ahmed et al. study did not explain any methods used for random sequence concealment and the risk of bias was judged to be unclear in this study [32]. Random sequence concealment was performed using sealed envelopes in Labban et al. study and this study was judged as low risk of bias [33]. In all three included studies, we judged the level of risk to be unclear for the item of ‘blinding participants and personnel’ [23, 32, 33]. Outcome assessors were blind in two studies and these studies were rated as low risk of bias [32, 33]. Blinding of outcome assessment was not performed in Al Amri et al. study and this study was judged to be high risk of bias [23]. None of the included studies presented a flow diagram with details about the withdrawals or loss to follow-up and were rated to be at unclear risk of bias [23, 32, 33]. All studies were free of selective reporting and the clinically important outcomes were reported by these studies. Thus, we rated these studies to be low risk of bias [23, 32, 33].

**Fig. 2a:**
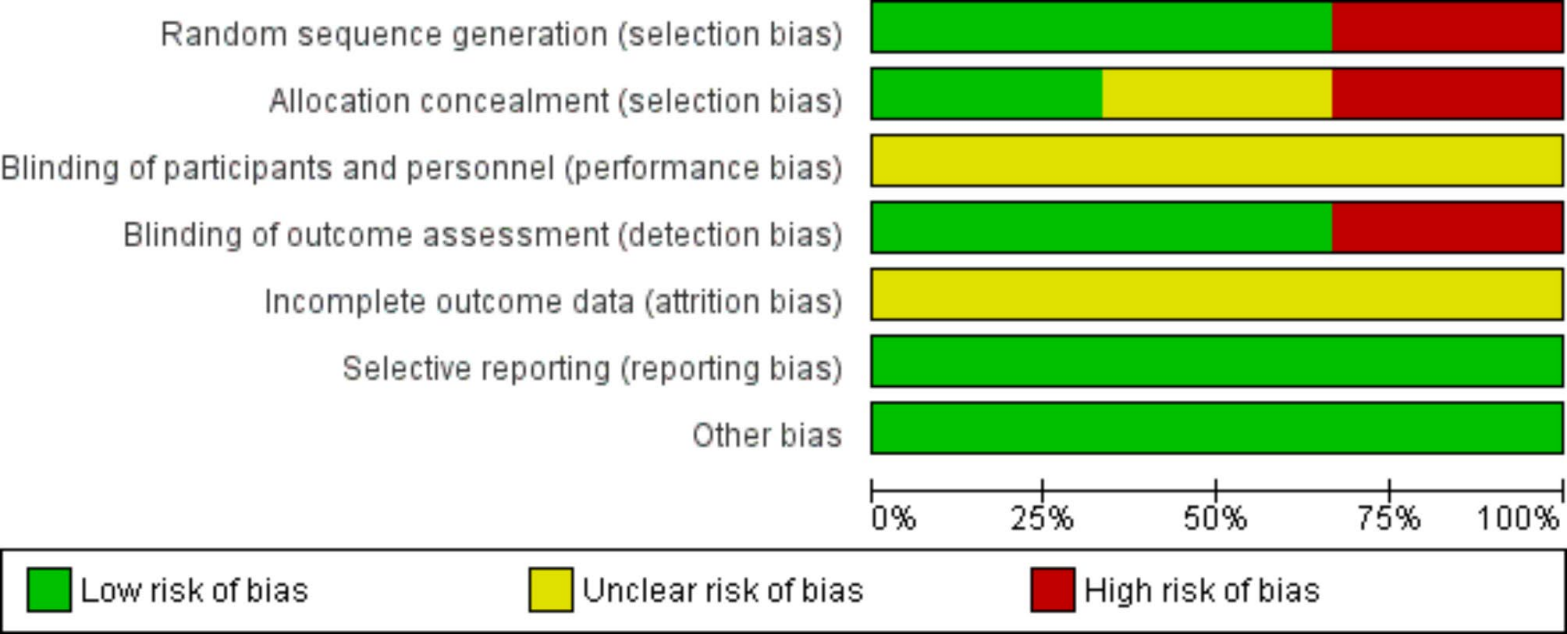
Risk of bias in the included

**Fig. 2b:**
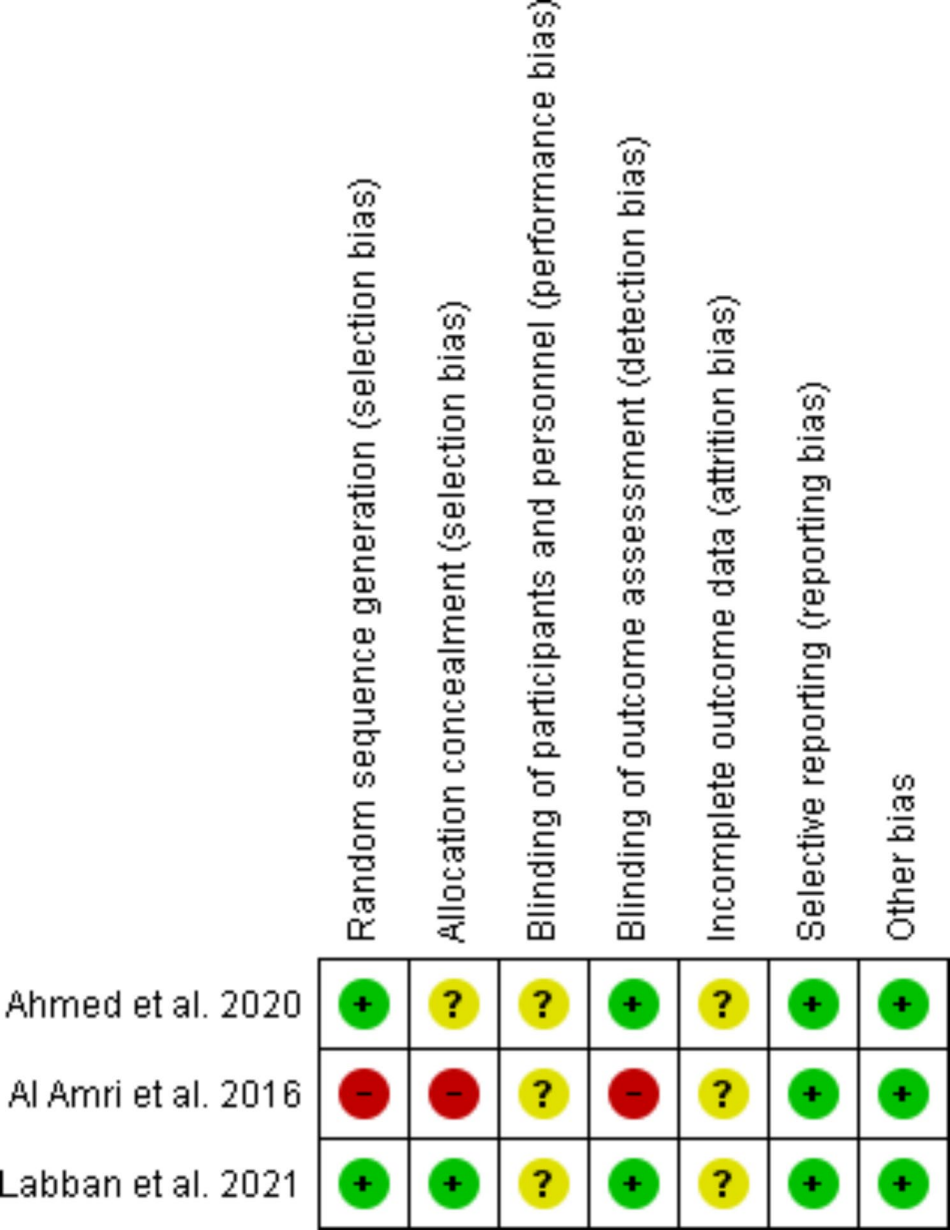
Review authors’ judgments about each risk of bias item for each included RCT

**Fig. 3:**
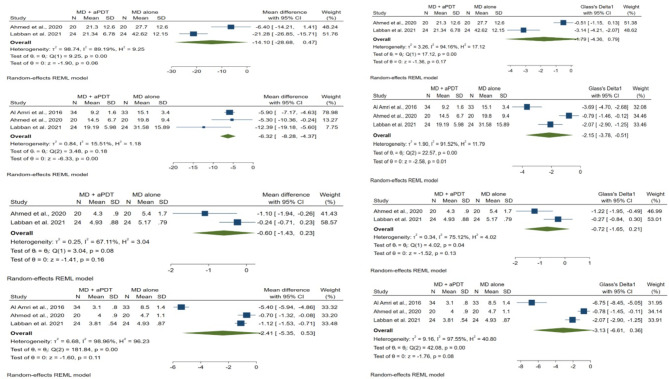
Forest plots for bleeding on probing and probing depth. Meta-analyses were carried out on the 3- and 6-month follow-up data

### Primary and secondary outcomes of the studies

**Fig. 4:**
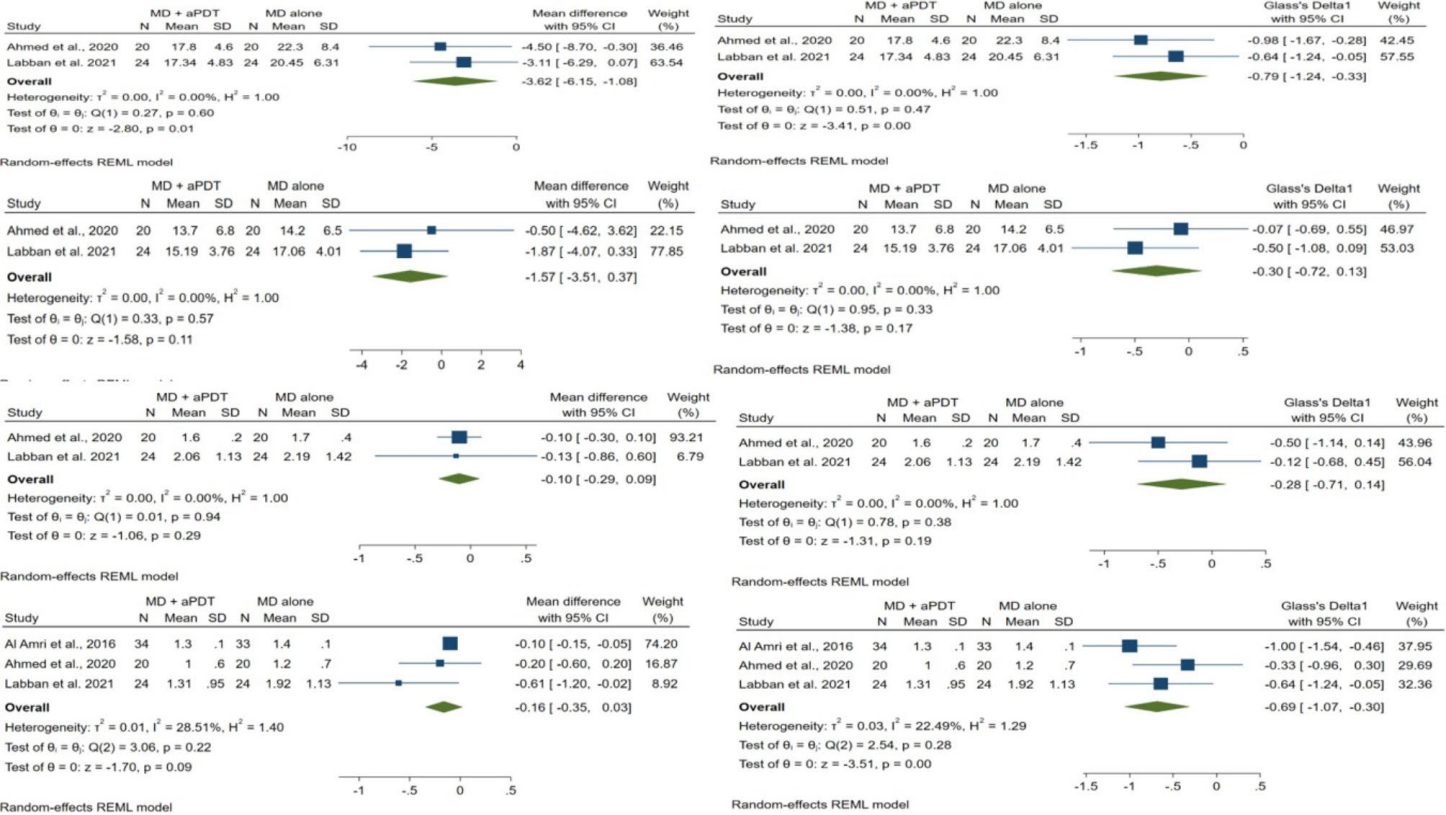
Forest plots for plaque index and crestal bone loss. Meta-analyses were carried out on the 3- and 6-month follow-up data

SMD of primary and secondary outcomes with 95% confidence intervals is presented in Figs. 3 and 4, respectively.

#### BOP

There was no significant difference between MD + a-PDT and MD alone groups in BOP at.

3-month follow-up (SMD = -1.79, 95% CI: -4.36 to 0.79, p = 0.17; two studies and Fig. 3) [32, 33]. However, a great amount of heterogeneity was found (*I*^*2*^ *=* 94.16%, p < 0.001). At 6-month follow-up, BOP was significantly lower in MD + a-PDT group in comparison with MD alone group (SMD = -2.15, 95% CI: -3.78 to -0.51, p = 0.01; three studies and Fig. 3) [23, 32, 33]. In addition, a great amount of heterogeneity was found (*I*^*2*^ *=* 91.52%, p < 0.001).

#### PD

PD did not differ significantly between the MD + a-PDT and MD alone groups (SMD = -0.72, 95% CI: -1.65 to 0.21, p = 0.13; two studies [32, 33], at 3-month, and SMD = -3.13, 95% CI: -6.61 to 0.36, p = 0.08; three studies [23, 32, 33] at 6-month follow-up, respectively, and Fig. 3). Furthermore, homogeneity assumption was not met (*I*^*2*^ *=* 75.12%, p = 0.04 at 3-month and *I*^*2*^ *=* 97.55%, p < 0.001 at 6-month, respectively).

#### PI

At 3-month follow-up, PI was significantly lower in MD + a-PDT group compared to that of MD alone group (SMD = -0.79, 95% CI: -1.24 to -0.33, p < 0.001; two studies and Fig. 4) [23, 32, 33]. No heterogeneity was observed (*I*^*2*^ *=* 0.00%, p = 0.47). At 6-month follow-up, the two groups were similar regarding PI (SMD = -0.30, 95% CI: -0.72 to 0.13, p = 0.17; two studies and Fig. 4) [23, 32, 33]. Furthermore, homogeneity assumption was met (*I*^*2*^ *=* 0.00%, p = 0.33).

#### CBL

No significant difference was revealed between the two groups regarding CBL at 3-month follow-up (SMD = -0.28, 95% CI: -0.71 to 0.14, p = 0.19; two studies and Fig. 4) [32, 33]. In addition, there was no heterogeneity between studies (*I*^*2*^ = 0.00%, p = 0.38). At 6-month follow-up, CBL was significantly lower in MD + a-PDT group in comparison with MD alone group (SMD = -0.69, 95% CI: -1.07 to -0.30, p < 0.001; three studies and Fig. 4) [23, 32, 33]. In addition, homogeneity assumption was satisfied (*I*^*2*^ = 22.49%, p = 0.28).

## Discussion

The present systematic review aimed to confirm whether a-PDT as an adjunct to MD is effective in improving clinical peri-implantitis parameters in patients with T2DM. The results indicated that a statistically significant reduction in PI at 3-month follow-up in MD + a-PDT group compared to MD group alone. In addition, MD + a-PDT group had significant improvements in BOP and CBL at 6-month follow-up. One explanation may be related to the fact that aPDT causes a significant reduction in the infiltration of inflammatory cells, including plasma cells and lymphocytes in the lamina propra of the subgingival connective tissue. Consequently, the elimination of these inflammatory cells contributes to a reduction in the beeding ratio [34]. Furthermore, this improvement of BOP could also be due to the presence of a photosensitizer in the depth of the pocket around the implant, which leads to the elimination of peri-implant pathogens. However, the MD + a-PDT group did not show any improvement in reducing PD, which may be related to the frequency of a-PDT use. It is not possible to maintain the antibacterial effect in low a-PDT sessions [35]. Overall, it can be speculated that if a-PDT is used as an adjunctive therapy to MD in in patients with peri-implantitis who have T2DM, the outcomes would have been significantly better as compared to MD alone. The reasons for this difference could be that adjuvant a-PDT has been shown to have a positive effect on microbiological parameters, improve connective tissue attachment, form more stable blood clots to support wound healing [36–38].

It can be noted from the included studies that some of the laser parameters were either missing or homogeneity assumption was not met [23, 32, 33]. Parameters related to laser therapy such as energy fluence, power density, and pre-irradiation time were lacking or different. Other parameters such as duration of irradiation, number of laser sessions, fiber diameter, and concentration of photosensitizer either varied considerably or were not reported in some studies. Evidence suggests that fiber diameter could impact the power density and energy output during a-PDT and can alter the actual amount of energy released during a-PDT, potentially affecting the antimicrobial efficacy [39]. Therefore, standardized laser parameters are necessary to interpret the efficacy of a-PDT in the management of periodontal parameters in T2DM.

The results of periodontal therapy are poorer in patients with poorly controlled T2DM than in patients with well-controlled T2DM [40]. The hyperglycemia can enhance tissues accumulation of advanced glycation end products (AGEs). The binding of AGEs to macrophage receptors leads to increased cytokine upregulation, such as interleukin-1 and tumor necrosis factor-a [41]. The formation of AGEs-collagen cross-link leads to impaired tissue integrity and increased susceptibility to pathogenic breakdown, as observed in T2DM patients with severe periodontal disease [42]. Stewart et al. indicated that glycemic control improved significantly in T2DM patients after periodontal therapy [43].

Strengths of our systematic review include a comprehensive review and sensitive search strategy, explicit inclusion and exclusion criteria, sensitivity analyses, and adherence to PRISMA guidelines to ensure a high quality report. The high heterogeneity among studies and the low number of included studies for analyses are the limitations of this study. Furthermore, a short follow up period (3–6 months) was reported in the included studies [32, 33]. In addition, the subgingival flora has been reported in patients with T2DM and periodontitis [44]. It is worth noting that most studies did not describe immunological-microbiological parameters after a-PDT treatment. These limitations may have influenced the outcomes assessed. The present systematic review suggests that a-PDT may help in improving periodontal parameters in peri-implantits patients with T2DM; however, more well-designed and large-scale RCTs that focus on clinical, microbiological and immunological parameters and exclude other confounding variables are also recommended. All these limitations should be considered when interpreting the present results. It is also recommended that further studies be conducted in T2DM patients with multiple a-PDT sessions to confirm its effects. In addition, for future studies, the authors suggest designing further studies with the current subject so that subgroup analyses can be conducted to find the source of heterogeneity.

## Conclusion

According to the meta-analysis, a-PDT can reduce PI at 3-month follow-up. Furthermore, BOP and CBL reduced at 6-month follow-up in patients with peri-implantitis who have T2DM. Also, it is recommended to perform more studies in T2DM patients with multiple sessions of a-PDT to validate its effects. In addition, considering the high heterogeneity of BOP and PD, the authors suggest for future RCTs to assess the efficacy of a-PDT + MD against periodontal parameters in T2DM patients.

### Electronic supplementary material

Below is the link to the electronic supplementary material.

Supplementary Material 1

## Data Availability

All data analyzed during this study are included in this published article.
