# Supplementary material for: Evaluating the effect of mechanical debridement with adjunctive antimicrobial photodynamic therapy in comparison with mechanical debridement alone on the peri-implant parameters in type 2 diabetic mellitus patients with peri-implantitis: a systematic review and meta-analysis
Source: BMC Oral Health. 2023 Oct 12;23:751. doi: 10.1186/s12903-023-03337-9 (PMC10571232; doi:10.1186/s12903-023-03337-9)
Supplement: Supplementary file 1 — Supplementary Material 1 [file 12903_2023_3337_MOESM1_ESM.docx]

| **Table S1** Search syntax in PubMed database |
| --- |
| ((“Peri-implantitis”[mh] OR Periimplantiti* OR “Peri-Implantiti*” OR “Peri Implantiti*” OR “Peri Implant disease*” OR “Periimplant disease*” OR “Peri Implant infection*” OR “Periimplant infection*” OR “Peri Implant inflammation*” OR “Periimplant inflammation*” OR “Peri Implant mucositi*” OR “Periimplant mucositi*”) AND (“Diabetes Mellitus, Type 2”[mh] OR “Diabetes Mellitus” OR NIDDM OR MODY OR “Maturity-Onset Diabetes” OR “Type 2 Diabetes” OR “Type ii Diabetes” OR “Diabetes Type 2” OR “Diabetes Type ii” OR “noninsulin dependent diabetes” OR “non-insulin dependent diabetes” OR “Ketosis-Resistant diabetes” OR t2dm OR “type 2 DM” OR “type ii DM” OR “DM type 2” OR “DM type ii” OR “slow-onset diabetes”) AND (Photochemotherapy[mh] OR “Lasers, Semiconductor”[mh] OR Photochemotherap* OR “Photodynamic Therap*” OR “photo chemo therap*” OR “photochemo therap*” OR “photo chemotherap*” OR chemoPhototherap* OR “chemo Phototherap*” OR “chemo Photo therap*” OR “chemo photo therap*” OR “light chemo therap*” OR “light chemotherap*” OR “Photo dynamic Therap*” OR “photodynamic treatment*” OR “photo dynamic treatment*” OR “laser dynamic therap*” OR (therap* AND photodynamic) OR  (therap* AND “photo dynamic”) OR “photodynamic process*” OR “photo dynamic process*” OR (process* AND photodynamic) OR (process* AND “photo dynamic”) OR PDT OR “Photo activated disinfection*” OR “Photoactivated disinfection*” OR “Photo activated chemotherap*” OR “Photoactivated chemotherap*” OR “Photo activated therap*” OR “Photoactivated therap*” OR (“Photo activated” AND therap*) OR (Photoactivated AND therap*) OR (“Photo thermal” AND therap*) OR (Photothermal AND therap*) OR “laser activated disinfection*” OR (“laser activated” AND disinfection*) OR “laser activated irrigation*” OR (“laser activated” AND irrigation*) OR “light activated disinfection*” OR (“light activated” AND disinfection*) OR “light activated irrigation*” OR (“light activated” AND irrigation*) OR “Photodynamic disinfection*” OR “Photo dynamic disinfection*” OR (Photodynamic AND disinfection*) OR “laser irradiat*” OR (laser AND irradiat*) OR “photo irradiat*” OR (photo AND irradiat*) OR “light irradiat*” OR (light AND irradiat*) OR “laser aided” OR “laser assisted” OR “Diode laser*” OR “Semiconductor Laser*” OR “Gallium Aluminum Arsenide Laser*” OR “GaAlAs Laser*” OR (laser AND GaAlAs) OR (Laser* AND “Gallium Aluminum Arsenide”) OR “low-level light therapy”[mh] OR “low level light therap*” OR (“low level” AND “light therap*”) OR “low-level laser therap*” OR (“low level” AND “laser therap*”) OR “low level photo therap*” OR “low level phototherap*” OR (“low level” AND “photo therap*”) OR (“low level” AND phototherap*) OR (“low-level light” AND therap*) OR (“low-level photo” AND therap*) OR (“low-level laser” AND therap*) OR “photobiomodulation therap*” OR “photobiomodulation treatment*” OR “photo biomodulation therap*” OR “photo biomodulation treatment*” OR “photobiostimulation therap*” OR “photobiostimulation treatment*” OR “photo biostimulation therap*” OR “photo biostimulation treatment*” OR “Laser Biostimulation” OR “photo Biostimulation” OR “photo Bio stimulation” OR photobiostimulation OR (Laser AND Biostimulation) OR (Laser AND “Bio stimulation”) OR (photo AND Biostimulation) OR (photo AND “Bio stimulation”) OR LLLT OR PBMT OR “low level laser irradiation” OR (“low level” AND “laser irradiation”) OR “low energy laser irradiation” OR (“low energy” AND “laser irradiation”) OR “low power laser irradiation” OR (“low power” AND “laser irradiation”) OR (“low power laser” AND irradiation) OR (“low level laser” AND irradiation) OR (“low energy laser” AND irradiation) OR “low level light irradiation” OR (“low level” AND “light irradiation”) OR “low energy light irradiation” OR (“low energy” AND “light irradiation”) OR “low power light irradiation” OR (“low power” AND “light irradiation”) OR (“low power light” AND irradiation) OR (“low level light” AND irradiation) OR (“low energy light” AND irradiation) OR “low level photo irradiation” OR (“low level” AND “photo irradiation”) OR “low energy photo irradiation” OR (“low energy” AND “photo irradiation”) OR “low power photo irradiation” OR (“low power” AND “photo irradiation”) OR (“low power photo” AND irradiation) OR (“low level photo” AND irradiation) OR (“low energy photo” AND irradiation) OR “low power light therap*” OR (“low power” AND “light therap*”) OR “low power laser therap*” OR (“low power” AND “laser therap*”) OR “low power photo therap*” OR (“low power” AND “photo therap*”) OR “low power laser therap*” OR (“low power” AND “laser therap*”) OR (“low power light” AND therap*) OR (“low power photo” AND therap*) OR (“low power laser” AND therap*) OR “low intensity light therap*” OR (“low intensity” AND “light therap*”) OR “low intensity laser therap*” OR (“low intensity” AND “laser therap*”) OR “low intensity photo therap*” OR (“low intensity” AND “photo therap*”) OR (“low intensity light” AND therap*) OR (“low intensity photo” AND therap*) OR (“low intensity laser” AND therap*) OR “laser photo therap*” OR “laser phototherap*” OR “photo therap*” OR phototherap* OR “soft laser therap*” OR (“soft laser” AND therap*) OR “cold laser therap*” OR (“cold laser” AND therap*) OR “cold laser” OR “soft laser” OR “low level light treatment*” OR “low level laser treatment*” OR “low level photo treatment*” OR (“low level” AND “light treatment”) OR (“low level” AND “laser treatment”) OR (“low level” AND “photo treatment”) OR "light emitting diode" OR (LED AND laser)) AND (“periodontal debridement”[mh] OR “subgingival curettage”[mh] OR “dental scaling”[mh] OR “root planing”[mh] OR “dental scaling”[mh] OR “dental prophylaxis”[mh] OR “dental polishing”[mh] OR “periodontal debridement*” OR “periodontal pocket debridement*” OR “pocket debridement*” OR “nonsurgical periodontal treatment*” OR (nonsurgical AND “periodontal treatment*”) OR “non-surgical periodontal treatment*” OR (“non-surgical” AND “periodontal treatment*”) OR (“periodontal pocket” AND debridement*) OR “implant debridement*” OR (debridement* AND implant*) OR “peri-implant debridement* OR “periimplant debridement* OR (debridement* AND “peri-implant*”) OR (debridement* AND periimplant*) OR “subgingival curettage*” OR “sub gingival curettage*” OR “gingival curettage*” OR (curettage* AND subgingival) OR (curettage* AND “sub gingival”) OR (curettage* AND gingival) OR “periodontal curettage*” OR (curettage* AND periodontal) OR “periimplant curettage*” OR “peri-implant curettage*” OR (curettage* AND periimplant) OR (curettage* AND “peri-implant”) OR “periodontal epithelial debridement*” OR “subgingival scaling*” OR (subgingival OR scaling*) OR (“sub-gingival” OR scaling*) OR (supragingival OR scaling*) OR (“supra-gingival” OR scaling*) OR (root OR scaling*) OR “sub-gingival scaling*” OR “supragingival scaling*” OR “supra-gingival scaling*” OR “root scaling*” OR “root planning*” OR “root planing*” OR “dental scaling*” OR (dental OR scaling*) OR “periodontal scaling*” OR (periodontal OR scaling*) OR “implant scaling*” OR (implant AND scaling*) OR “periimplant scaling*” OR (periimplant AND scaling*) OR “peri-implant scaling*” OR (“peri-implant” AND scaling*) OR (dental AND prophylaxis) OR “periodontal prophylaxis” OR (periodontal AND prophylaxis) OR “implant prophylaxis” OR (implant AND prophylaxis) OR “peri-implant prophylaxis” OR (“peri-implant” AND prophylaxis) OR “periimplant prophylaxis” OR (periimplant AND prophylaxis) OR “subgingival prophylaxis” OR “sub-gingival prophylaxis” OR (subgingival AND prophylaxis) OR (“sub-gingival” AND prophylaxis) OR (supragingival AND prophylaxis) OR (“supra-gingival” AND prophylaxis) OR “supra-gingival prophylaxis” OR “supragingival prophylaxis” OR “dental cleaning” OR (cleaning AND dental) OR “periodontal cleaning” OR (periodontal AND cleaning) OR “subgingival cleaning” OR “sub-gingival cleaning” OR (subgingival AND cleaning) OR (“sub-gingival” AND cleaning) OR (supragingival AND cleaning) OR (“supra-gingival” AND cleaning) OR “supra-gingival cleaning” OR “supragingival cleaning” OR “implant cleaning” OR (implant AND cleaning) OR “peri-implant cleaning” OR (“peri-implant” AND cleaning) OR “periimplant cleaning” OR (periimplant AND cleaning) OR “mouth debridement*” OR “mouth disinfection” OR (mouth OR disinfection) OR “pocket disinfection” OR (pocket OR disinfection) OR “dental disinfection” OR (dental OR disinfection) OR “periodontal disinfection” OR (periodontal OR disinfection) OR “implant disinfection” OR (implant OR disinfection) OR “subgingival disinfection” OR (subgingival OR disinfection) OR “sub-gingival disinfection” OR (“sub-gingival” OR disinfection) OR “supragingival disinfection” OR (supragingival OR disinfection) OR “supra-gingival disinfection” OR (“supra-gingival” OR disinfection) OR “gingival disinfection” OR (gingival OR disinfection) OR “plaque removal” OR (plaque OR removal) OR “dental polishing” OR (dental OR polishing) OR “dental finishing” OR “dental burnishing” OR “implant polishing” OR (implant OR polishing) OR “mechanical debridement*” OR “dental cleaning procedure*”)) OR ((“Peri-implantitis”[mh] OR Periimplantiti* OR “Peri-Implantiti*” OR “Peri Implantiti*” OR “Peri Implant disease*” OR “Periimplant disease*” OR “Peri Implant infection*” OR “Periimplant infection*” OR “Peri Implant inflammation*” OR “Periimplant inflammation*” OR “Peri Implant mucositi*” OR “Periimplant mucositi*”) AND (“Diabetes Mellitus, Type 2”[mh] OR “Diabetes Mellitus” OR NIDDM OR MODY OR “Maturity-Onset Diabetes” OR “Type 2 Diabetes” OR “Type ii Diabetes” OR “Diabetes Type 2” OR “Diabetes Type ii” OR “noninsulin dependent diabetes” OR “non-insulin dependent diabetes” OR “Ketosis-Resistant diabetes” OR t2dm OR “type 2 DM” OR “type ii DM” OR “DM type 2” OR “DM type ii” OR “slow-onset diabetes”) AND (Periodontal Index[mh] OR “Periodontal Index*” OR “Periodontal Indices” OR (Index* AND Periodontal) OR (Indices AND Periodontal) OR “Bleeding on Probing” OR BOP OR “Probing pocket depth” OR “pocket probing depth” OR PPD OR “probing depth” OR (Probing AND “pocket depth”) OR “CPITN” OR “gingival index*” OR “gingival indices” OR Dental Plaque Index[mh] OR “Plaque Index*” OR “Plaque Indices” OR (Index* AND Plaque) OR (Indices AND Plaque) OR “plaque score*” OR (score* AND Plaque) OR “Periimplant bleeding” OR “Peri implant bleeding” OR (periimplant AND bleeding) OR (“peri implant” AND bleeding) OR Gingival Hemorrhage[mh] OR “Gingival Hemorrhage*” OR (hemorrhage* AND gingiva) OR “sulcus bleeding” OR (sulcus AND bleeding) OR “gingival bleeding” OR (bleeding AND gingiva*) OR “sulcus bleeding” OR “bone gain” OR (bone AND gain) OR “Alveolar Bone Loss”[mh] OR “Alveolar Bone Loss*” OR “Alveolar Process Atroph*” OR “Alveolar Resorption*” OR (Resorption* AND Alveolar) OR (“Bone Loss*” AND Periodontal) OR “Periodontal Bone Loss*” OR “Periodontal Resorption*” OR (Resorption* AND periodontal) OR “Alveolar Bone Atroph*” OR (“bone atroph*” AND Alveolar) OR (“Bone Loss*” AND alveolar) OR “radiographic bone loss” OR “Periodontal Attachment Loss”[mh] OR (“Attachment Loss” AND Periodontal) OR “clinical attachment loss” OR (“bone resorption”[mh] AND periodontal) OR (“bone resorption”[mh] AND alveo*) OR “crestal bone loss”)) |
